# Spontaneous improvement of sarcoidosis mimicking a vertebral metastatic lesion: It's indeed possible!

**DOI:** 10.1002/ccr3.3172

**Published:** 2020-07-22

**Authors:** Safa Rahmouni, Kaouther Maatallah, Hanene Ferjani, Hend Riahi, Lamia Manamani, Dhia Kaffel, Wafa Hamdi

**Affiliations:** ^1^ Rheumatology Department Kassab Orthopedics Institute Ksar Saïd Tunisia; ^2^ Radiology Department Kassab orthopedics institute Ksar Saïd Tunisia; ^3^ Rheumatology Department Mahmoud El Matri Hospital Ariana Tunisia

**Keywords:** bone metastasis, magnetic resonance imaging, vertebral sarcoidosis

## Abstract

The diagnosis of vertebral sarcoidosis can be challenging. Besides, there is no consensus for the treatment of these lesions. Exceptionally, lesions may show spontaneous regression. To our knowledge, there are only two case reports of spontaneous resolution of vertebral sarcoidosis documented by follow‐up MRI. Herein, we present a third case.

## Graphical abstract

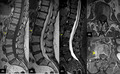

## 1 INTRODUCTION

Vertebral sarcoidosis is a systemic inflammatory granulomatous disease that can develop in almost any organ system. In rare cases, it can affect the bones. Vertebral sarcoidosis is even rarer. MRI represents a fundamental tool for the assessment of vertebral involvement. However, sarcoidosis‐related bone lesions can be similar to bone metastases, making the diagnosis rather challenging. Therefore, performing a biopsy is important in order to confirm the diagnosis. There is no consensus for the treatment of vertebral sarcoidosis. In very rare cases, lesions may show spontaneous regression on follow‐up images. To our knowledge, there are only two case reports of spontaneous resolution of vertebral sarcoidosis documented by follow‐up MRI.^1^, ^2^

Herein, we present a case of vertebral sarcoidosis mimicking osseous metastases that resolved spontaneously in a 65‐year‐old woman.

## 2 CASE REPORT

In 2018, a 65‐year‐old white woman presented with various symptoms including 2 years of gradually worsening mechanical low back pain and deterioration of the general status.

She had hypertension and hypertensive renal disease. She had a known history of stage 2 pulmonary sarcoidosis for 7 years. Evidence supporting the diagnosis of sarcoidosis included erythema nodosum, chronic arthritis lymphopenia, and hilar and mediastinal lymphadenopathy. The disease seemed inactive given that the patient never had sarcoidosis‐related symptoms nor received treatment throughout the years.

Physical examination showed no abnormalities except for lower lumbar spinous percussion tenderness. There were no skin lesions nor peripheral lymphadenopathy. Routine laboratory tests showed creatinine clearance of 48.5 ml/min, hypercalcemia of 2.86 mmol/l, normal serum phosphate concentration of 1.19 mmol/l, and normal liver enzymes and an erythrocyte sedimentation rate of 66 mm/h. Protein electrophoresis revealed hypoalbuminemia of 33.9 g/l and polyclonal hypergammaglobulinemia of 22.1 g/L.

**Figure 1:**
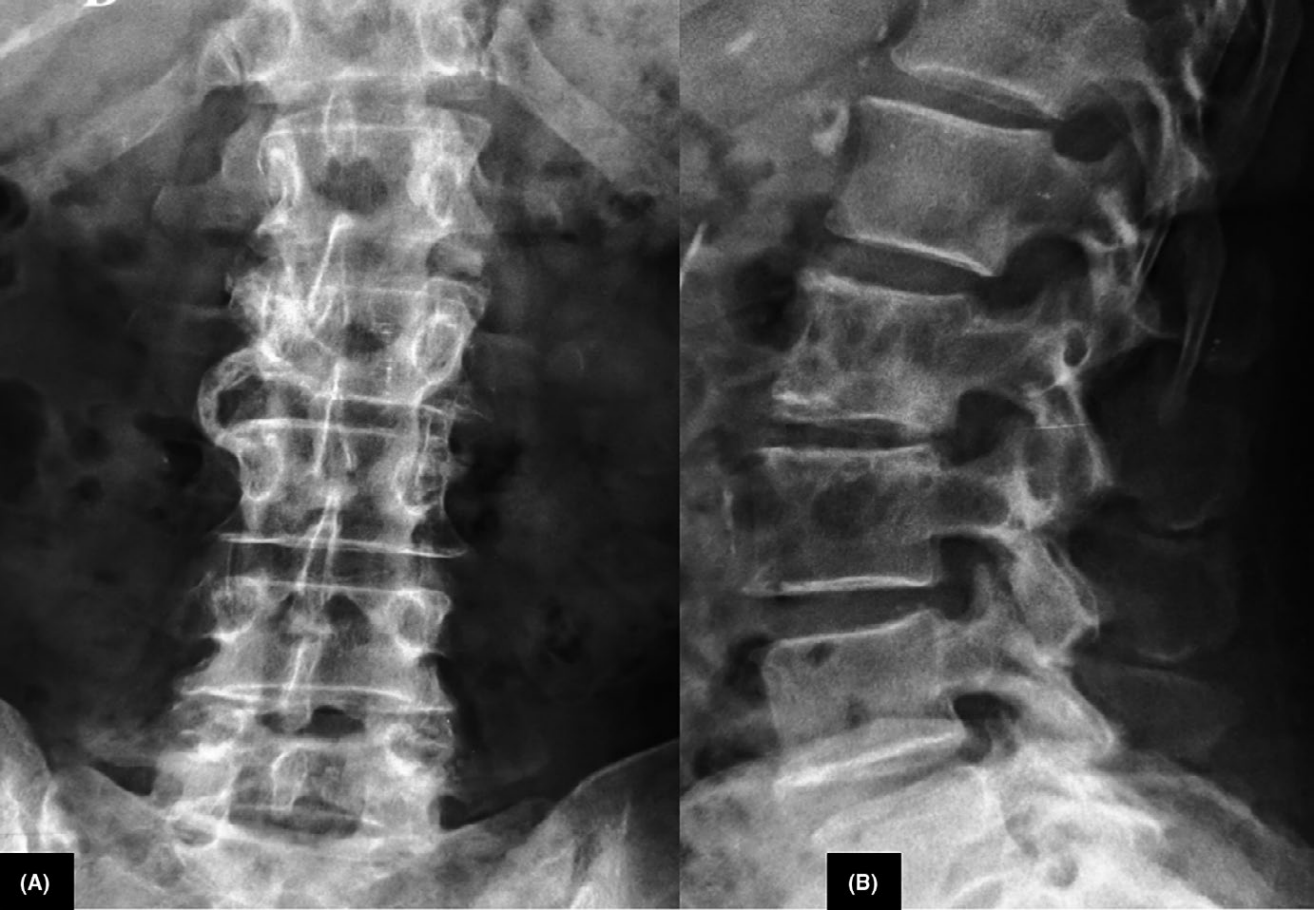
Anteroposterior (A) and lateral (B) radiographs of lumbar spine: mixed lytic‐sclerotic pattern in the L2 vertebra

**Figure 2:**
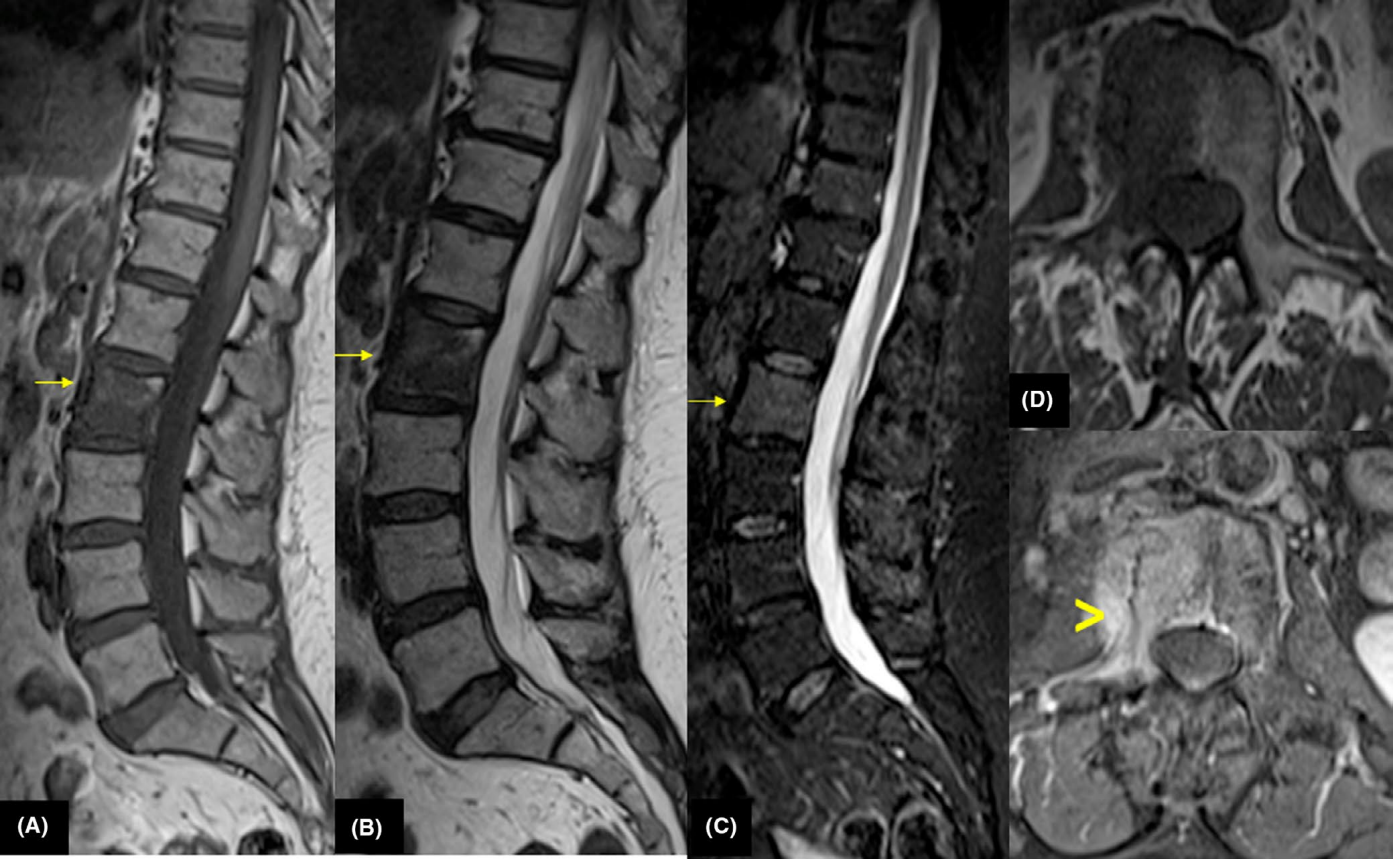
MRI sagittal T1 WI (A), sagittal T2 WI (B), sagittal STIR WI (C), axial T1 WI (D), and axial T1 fat sat gadolinium show decreased signal on T1 WI and high signal on T2 and after gadolinium administration throughout vertebral bodies (arrow) with soft‐tissue mass (arrowhead)

A lumbar plain radiograph showed a mixed lytic‐sclerotic pattern with a rim of sclerosis around the central osteolysis in the L2 vertebra (Figure 1). MRI demonstrated low signal intensity lesions in L2 on T1‐weighted (T1 WI) and high signal intensity on T2‐weighted (T2 WI) images with extension into the posterior elements. Lesions displayed high signal intensity on short T1 inversion‐recovery (STIR) images and enhancement after IV gadolinium administration (Figure 2). There was paravertebral soft‐tissue involvement without radiological signs of extension into the epidural space nor cord compression.

**Figure 3:**
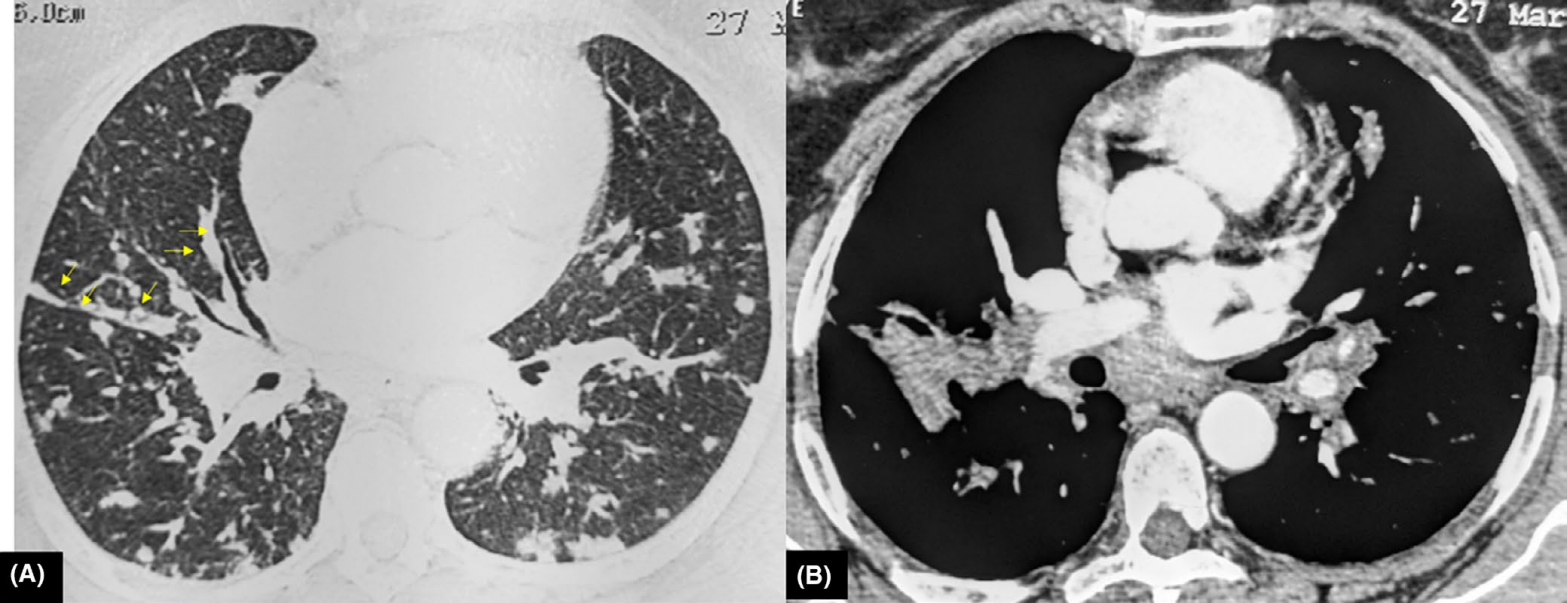
Axial CT of the chest (A, B) shows small nodules in a perilymphatic distribution along interlobular septa and the peribronchovascular (arrows)

**Figure 4:**
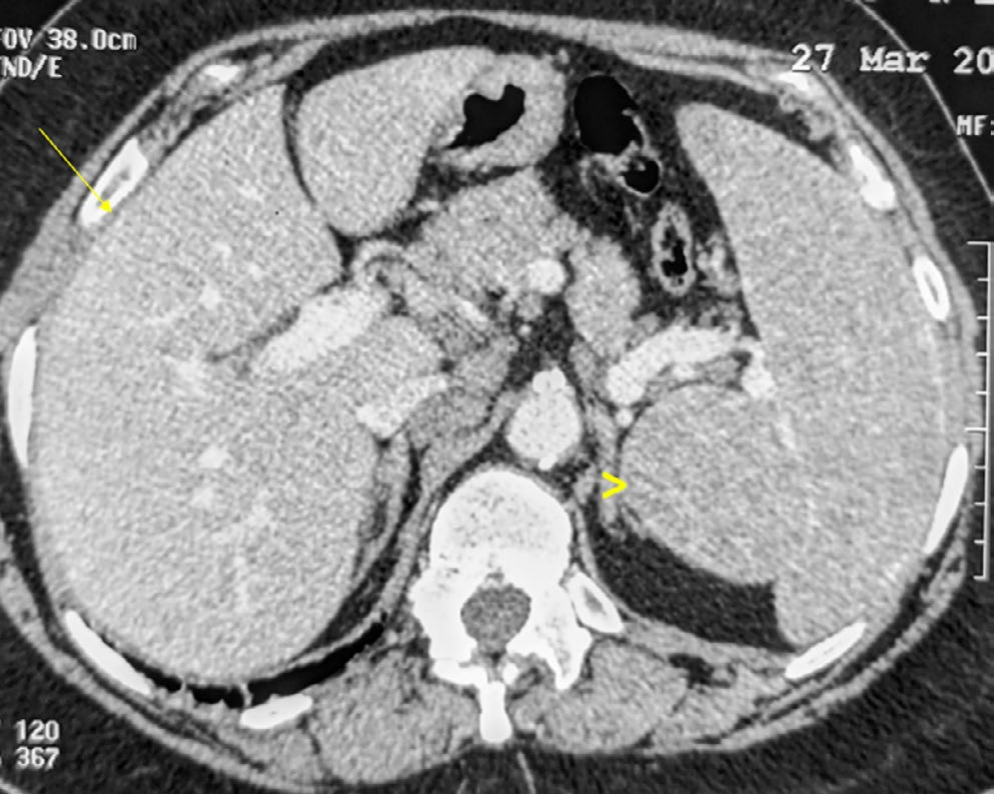
Axial CT of the abdomen shows hepatomegaly (arrow) and splenomegaly (arrowhead)

**Figure 5:**
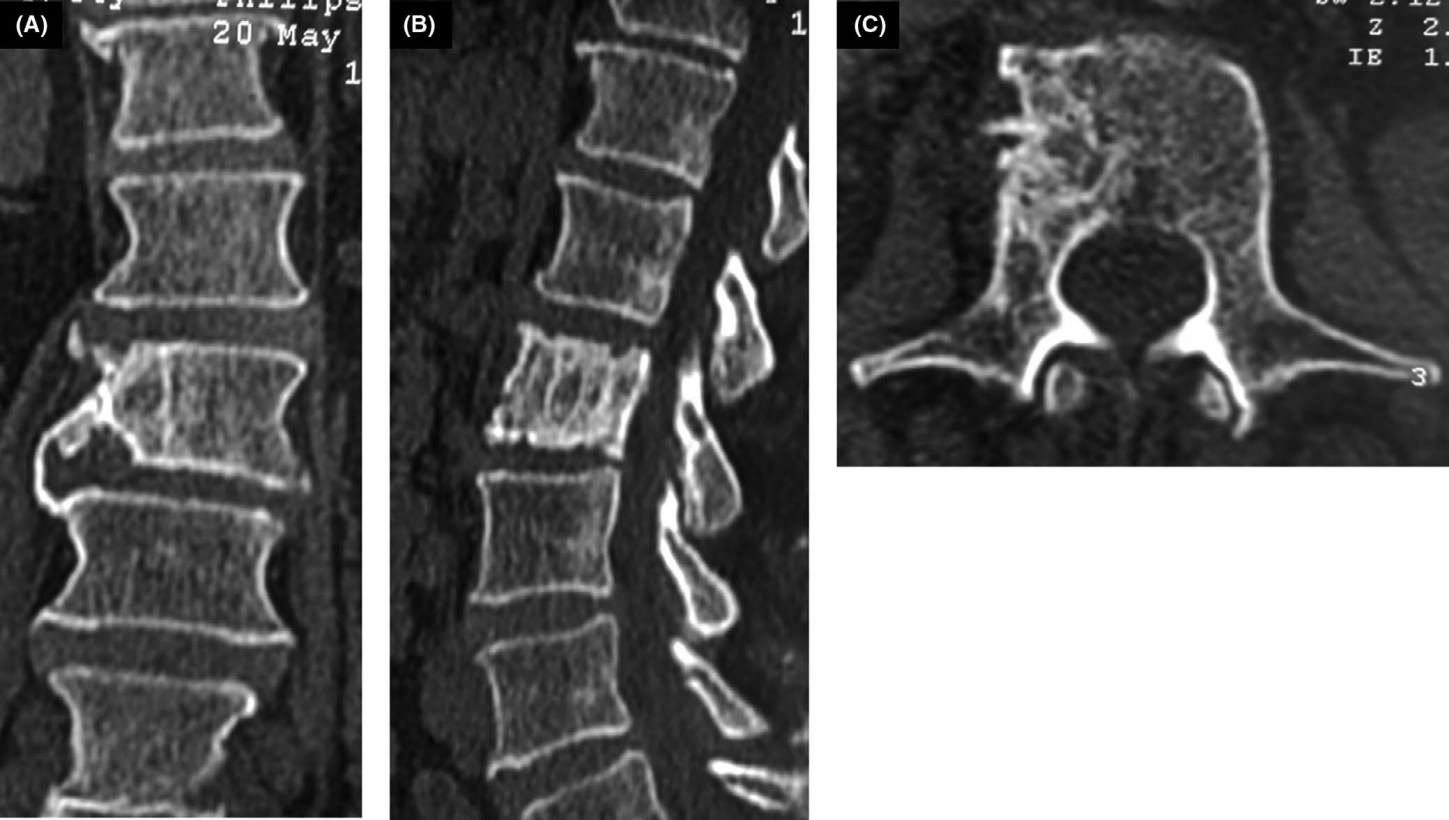
Coronal (A), sagittal (B), and axial (C) CT scans show sclerotic lesion of L2 vertebra with central osteolysis, an extension to the pedicle and cortical destruction

**Figure 6:**
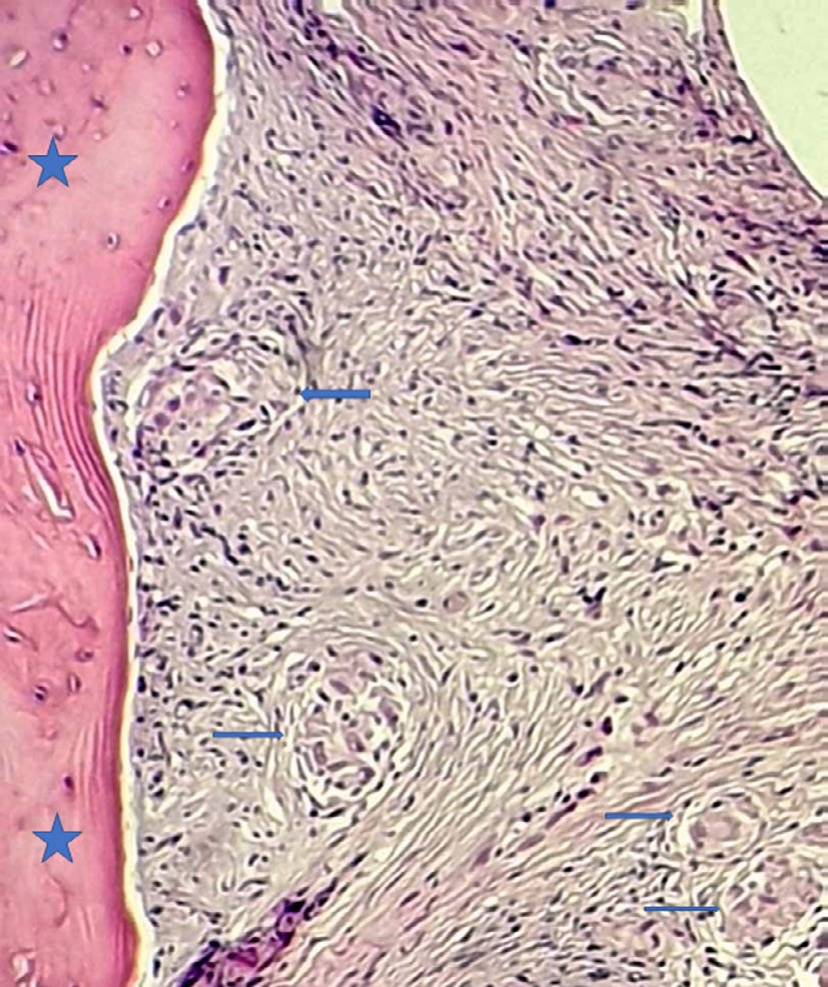
Biopsy of the L2 vertebra: The lesion showed bone trabeculae (stars) surrounded by fibrous stroma which is containing multiple well‐formed, noncaseating epithelioid granulomas (arrows) composed of multinucleated giant cells, epithelioid histiocytes, and scattered lymphocytes (hematoxylin and eosin, original magnification ×200)

Given the deterioration of the general status associated with bone lesions, the patient underwent extensive screening for hematologic and solid tumor malignancies including mammogram, neck ultrasound, and CT scans of the chest, abdomen, and pelvis. CT scans showed bilateral lung nodules (Figure 3), parenchymal condensation, bilateral hilar and mediastinal adenopathy, enlarged spleen measuring 14.5 cm, and hepatomegaly (Figure 4). CT images of the bone window revealed a sclerotic lesion in L2 vertebra with central osteolysis, an extension to the pedicle and cortical destruction (Figure 5), as well as sclerosis and irregularities of the left sacroiliac joint margins. The patient underwent a percutaneous CT‐guided biopsy of the L2 vertebra. The biopsy demonstrated noncaseating granulomata with no evidence of malignancy (Figure 6).

The diagnosis of systemic sarcoidosis with osseous involvement was established, and the patient was prescribed steroid treatment at the dose of 60 mg, however not taken because of the lack of resources.

She was lost to follow‐up for 1 year. At that point, she was readmitted to our hospital with a chief complaint of mechanical low back pain. A follow‐up CT scans at 1 year showed the persistence of the pulmonary nodules, the parenchymal condensation, the bilateral mediastinal adenopathy, and the enlarged spleen, in addition to the appearance of hepatic nodules.

**Figure 7:**
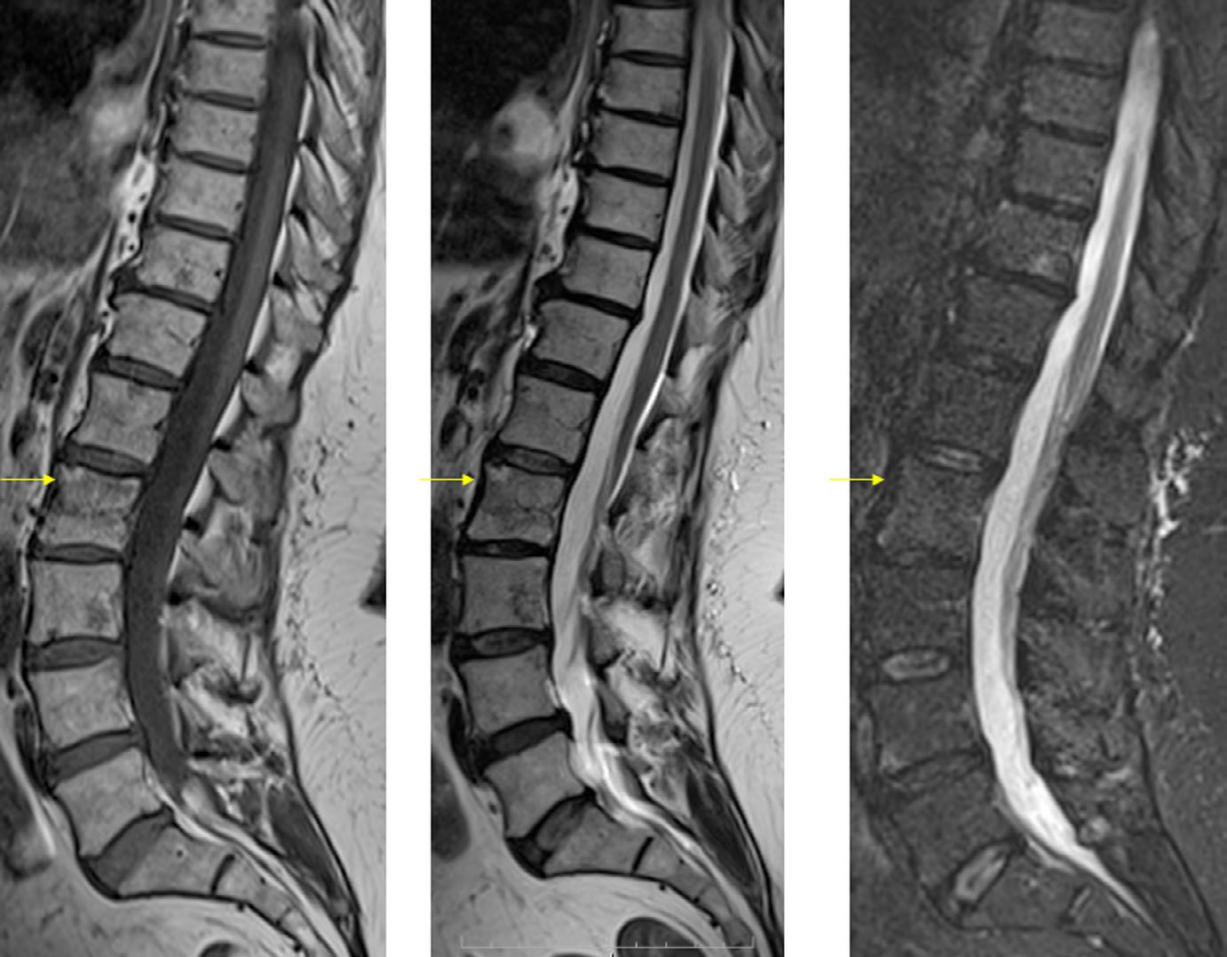
Follow‐up MRI shows almost complete disappearance of bone marrow abnormalities

Repeated MRI revealed almost complete resolution of the areas of marrow signal abnormality and the disappearance of the paravertebral soft‐tissue involvement (Figure 7). Furthermore, MRI demonstrated the L4‐L5 degenerative disk disease.

The patient was prescribed steroids in the form of oral prednisolone given the multisystem involvement.

## 3 DISCUSSION

Sarcoidosis is an inflammatory granulomatous condition occurring more commonly in women and black individuals. The lungs are involved in more than 90% of cases. Abdominal viscera are commonly involved in sarcoidosis, although this involvement is usually asymptomatic. Symptomatic hepatic sarcoidosis occurs in less than 10% of patients.^3^ Elevated liver enzymes (ALP and GGT) are the most common presentation. On imaging, hepatomegaly and hypodense nodular lesions are the hallmark features. Our patient had asymptomatic hepatomegaly with normal liver enzymes. Splenic involvement is reported in up to 40%‐60% of patients. It usually presents as either splenomegaly as observed in our patient or, less commonly, as multiple focal splenic lesions with no symptoms or rarely systemic constitutional symptoms.^4^

Osseous involvement is much less frequent with a variable prevalence ranging from 3% to 13% depending on the diagnostic modalities.^5^ Spinal sarcoidosis is even rarer and usually involves the thoracolumbar region. Our patient had multisystem involvement with pulmonary liver, spleen, and thoracic and extra‐thoracic lymph node adenopathy. This observation is consistent with the previous studies which demonstrated that osseous involvement is associated with long‐standing severe sarcoidosis with multi‐organ involvement.^6^

Patients with vertebral lesions usually report mechanical pain or tenderness over the involved area. Clinical presentation is nonspecific, and backache or radicular pain can reveal the disease. In addition to the sarcoidosis‐related bone lesions, our patient had degenerative disk disease. The latter could be responsible for the symptomatology.

The lesions can be lytic, sclerotic, or mixed lytic and sclerotic. The posterior elements and intervertebral disks are usually spared. However, an extension to the pedicle as demonstrated in our patient is possible and has been previously reported.^7^

On MRI, the lesions can have a variety of appearances. Typical MRI features include multifocal lesions within the vertebrae that have low signal intensity on T1‐weighted images, high signal intensity on T2‐weighted images, and variable enhancement following contrast medium administration depending on the nature of the tissue.^8^ Comparable findings were reported in our patient.

In addition, MRI provides detailed information about an extension to soft tissues, as demonstrated in our patient.^9^ However, MRI findings may vary widely and are not specific. The MRI appearance of vertebral sarcoidosis can be similar to osseous metastases.^10^

Before the bone biopsy, many differential diagnoses should be discussed, including tuberculosis, metastasis, multiple myeloma, lymphoma, eosinophilic granuloma, and disseminated echinococcal lesion.

The osseous lesions in sarcoidosis generally present without hypercalcemia. However, our patient had abnormal serum calcium level. This finding raised concerns about possible malignancy. Given the possible coexistence of sarcoidosis and metastases,^11^ an extensive screening for malignancy was conducted. In this case, the enlarged spleen, adenopathy (mediastinal and retroperitoneal), the bone lesions, and the extension to paravertebral were highly suggestive of lymphoma. Our patient was carefully screened for tuberculosis given the presence of constitutional symptoms and pulmonary nodules. Besides, tuberculosis is still endemic in our country. However, the intradermal tuberculin test was negative and the absence of caseation necrosis did not support this diagnosis.

As previously suggested, performing a biopsy is the key to confirm the diagnosis of vertebral sarcoidosis. Noncaseating granuloma is the hallmark of this disease.

Besides vertebral lesions, our patient had sacroiliitis. However unusual, the prevalence of sacroiliitis in sarcoidosis varies from 6% to 23.4%.^12^

Consistent with the previous studies, our patient had unilateral involvement of sacroiliac joints with typical imaging findings of sclerosis and joint margin irregularities. For reminder, our patient did not present with the typical inflammatory back pain history, and the pain was rather mechanical.

Given its rarity, there is no consensus for the treatment of osseous sarcoidosis. While asymptomatic patients do not require treatment, patients with pain and bone destruction should be treated. In our case, and given the multisystem involvement, steroids had been prescribed. Oral corticosteroids are the cornerstone of the treatment. They have been proven successful in reducing both symptoms and radiological changes.^13^ However, our patient did not receive the treatment prescribed for her.

The follow‐up MRI showed almost complete regression of bone marrow abnormality. To our knowledge, only two previous studies have reported spontaneous radiological resolution of vertebral sarcoidosis.^1^, ^2^

We also confirmed the absence of radioclinical parallelism as our patient remained symptomatic despite the radiological resolution of the skeletal lesions.^6^

Another possible explanation of the low back pain would be the L4‐L5 degenerative disk disease demonstrated on follow‐up MRI.

This case highlights the limitations of MRI to distinguish osseous sarcoidosis from metastatic lesions, the importance of considering vertebral involvement in a patient with a history of sarcoidosis, and the need for biopsy to ensure the accurate diagnosis of vertebral lesions.

Our case is unique and instructive for several reasons. The first is the coexistence of pulmonary, splenic, hepatic, vertebral, and sacroiliac involvement in the same patient. The second is the evidence of almost complete spontaneous resolution of widespread osseous documented by follow‐up MRI, which is rarely reported. And lastly, this report emphasizes the utility of follow‐up MR imaging to document treatment response given the lack of radioclinical parallelism.

**KEY POINTS**

Vertebral sarcoidosis should be kept in mind when exploring osseous pain in patients with documented sarcoidosis. MRI appearance can be similar to that of bone metastases.
Spontaneous improvement of radiographic findings is very rare but possible.

## CONFLICT OF INTEREST

None declared.

## AUTHOR CONTRIBUTIONS

SR: drafted the manuscript with the help of KM. HF: helped us to make the final diagnosis and participated in writing the text. HR: took the pictures and helped with the diagnosis. LM: was the first doctor who examined the patient, and helped with the final diagnosis. DK: revised the manuscript. WH: approved the final manuscript.

## ETHICAL APPROVAL

Ethical approval for this study was obtained from the Scientific and Ethical Committees of the hospital.

## Data Availability

The datasets used and/or analyzed during the current study are available from the corresponding author on reasonable request.
